# Role of radiomics as a predictor of disease recurrence in ovarian cancer: a systematic review

**DOI:** 10.1007/s00261-024-04330-8

**Published:** 2024-05-15

**Authors:** Niall J. O’Sullivan, Hugo C. Temperley, Michelle T. Horan, Waseem Kamran, Alison Corr, Catherine O’Gorman, Feras Saadeh, James M. Meaney, Michael E. Kelly

**Affiliations:** 1 Department of Radiology, St. James’s Hospital, Dublin, Ireland; 2 School of Medicine, Trinity College Dublin, Dublin, Ireland; 3 The National Centre for Advanced Medical Imaging (CAMI), St. James’s Hospital, Dublin, Ireland; 4 Department of Surgery, St. James’s Hospital, Dublin, Ireland; 5 Department of Gynaecology, St. James’s Hospital, Dublin, Ireland

**Keywords:** Radiomics, Radiogenomics, Oncology, Ovarian cancer, Recurrence

## Abstract

Ovarian cancer is associated with high cancer-related mortality rate attributed to late-stage diagnosis, limited treatment options, and frequent disease recurrence. As a result, careful patient selection is important especially in setting of radical surgery. Radiomics is an emerging field in medical imaging, which may help provide vital prognostic evaluation and help patient selection for radical treatment strategies. This systematic review aims to assess the role of radiomics as a predictor of disease recurrence in ovarian cancer. A systematic search was conducted in Medline, EMBASE, and Web of Science databases. Studies meeting inclusion criteria investigating the use of radiomics to predict post-operative recurrence in ovarian cancer were included in our qualitative analysis. Study quality was assessed using the QUADAS-2 and Radiomics Quality Score tools. Six retrospective studies met the inclusion criteria, involving a total of 952 participants. Radiomic-based signatures demonstrated consistent performance in predicting disease recurrence, as evidenced by satisfactory area under the receiver operating characteristic curve values (AUC range 0.77–0.89). Radiomic-based signatures appear to good prognosticators of disease recurrence in ovarian cancer as estimated by AUC. The reviewed studies consistently reported the potential of radiomic features to enhance risk stratification and personalise treatment decisions in this complex cohort of patients. Further research is warranted to address limitations related to feature reliability, workflow heterogeneity, and the need for prospective validation studies.

**Supplementary Information:**

The online version contains supplementary material available at 10.1007/s00261-024-04330-8.

## Introduction

Ovarian cancer is a significant health concern worldwide, accounting for 4.4% of entire cancer-related mortality in women [1, 2]. High mortality rates can be attributed to late-stage diagnosis, limited treatment options, and disease recurrence seen in 80% of advanced cancers, with no recommended screening strategy in place [3]. Traditional prognostic factors, such as histological subtype and tumour stage, have provided insight into patient outcomes; however, there is a growing need for more accurate and personalised prognostic tools to guide treatment decisions and improve overall patient management [4–6].

Radiomics, a rapidly evolving field within medical imaging, holds great promise in enhancing the prognostic evaluation of ovarian cancer [7]. Following acquisition of images, raw imaging data must be pre-processed via manual, semi-automated, or fully automated machine learning methods to facilitate segmentation of the regions of interest (ROI) [8–10]. Radiomic features can then be extracted from the ROIs using various feature extraction software, such as PyRadiomics, Computational Environment for Radiological Research (CERR), and Image Biomarker Explorer (IBEX) [11–14]. Specific features can be broadly subdivided into textural, morphological, and functional radiomics [15]. By extracting and analysing quantitative features from medical images, radiomics offers the potential to uncover hidden patterns and relationships that can serve as predictive markers [16]. In the context of ovarian cancer, radiomics can provide valuable insights into tumour heterogeneity, microenvironment characteristics, and treatment response [17, 18]. The integration of radiomics-based predictive models into clinical practice has the potential to facilitate individualised treatment strategies and improve patient outcomes [19].

Whilst several studies have investigated the role of radiomics in predicting disease recurrence in various cancer subtypes with promising performance, its specific application in ovarian cancer is an area of ongoing research [20–23]. The aim of our systematic review is to comprehensively assess the existing literature regarding the role of radiomics as a predictor of disease recurrence in ovarian cancer. Moreover, we aim to explore the potential clinical implications and future directions of radiomics in the management of ovarian cancer, ultimately laying the groundwork for future research to the development of more effective and personalised treatment strategies.

## Methods

### Study design and reporting guidelines

This study is a systematic review of retrospective studies and follows the Preferred Reporting Items for Systematic Reviews and Meta-Analyses (PRISMA) reporting guidelines. [24]

### Search strategy

The following databases were searched as part of the systematic review in August 2023: Medline, EMBASE, and Web of Science. The systematic search process with detailed search terms are outlined in the supplementary material S1. The last date of search was 11th August 2023. The grey literature (conference abstracts and dissertations) was also searched to further identify other suitable publications.

### Eligibility criteria

Studies were assessed for eligibility based on the following inclusion criteria. Studies investigating the use of radiomics to predict post-operative recurrence in patients undergoing primary debulking surgery with ovarian cancer were included in our analysis. Case reports, case series and conference abstracts were excluded. Radiomics features extracted from computed tomography (CT) and magnetic resonance imaging (MRI) were included.

### Study selection, data extraction & critical appraisal

A database was created using the reference managing software EndNote X9™. Two researchers (NOS and HCT) reviewed outputs from the searches independently of each other.

Initially, duplicates were removed. Study titles were then screened and assessed for potential relevance. The abstracts of selected potential studies were then read and assessed for eligibility for inclusion, based on the inclusion/exclusion criteria detailed above. Rejected studies were grouped together in the database by their reason for exclusion. The full texts of the abstracts deemed eligible for inclusion were then further analysed using the same criteria.

In order to extract and store data efficiently, the Cochrane Collaboration screening and data extraction tool, Covidence, was used [25]. Data were collected by two reviewers (NOS and HCT) independently, using the following headings; study details, study design, population, intervention, comparison groups and outcomes. Conflicts on study selection and data extraction between the two reviewers (NOS and HT) were resolved following an open discussion and final decision by senior author (MK).

A critical appraisal of the methodological quality and risk of bias of the included studies was performed. The critical appraisal was completed by two reviewers independently. Quality assessment of the included studies was performed according the Quality Assessment of Diagnostic Accuracy Studies 2 (QUADAS-2) and Radiomics Quality Score (RQS) [26, 27]. A description of these tools, including methodological domains, is provided for the reader in the supplementary material.

### Systematic review registration

Our systematic review was registered on PROSPERO in July 2023 (ID: CRD42023446290). [28]

### Statistical analysis

Due to heterogeneity in primary outcome, a meta-analysis of included studies was not performed. Data have been presented qualitatively throughout the results.

## Results

### Search results

The literature search described above yielded a total of 295 results (supplementary material S1). Following the removal of 103 duplicates, 192 studies were screened. After the initial screen, 86 abstracts were reviewed and assessed for eligibility, of which 15 were selected for full-text review. From these fifteen full texts, a total of six studies met the inclusion criteria and were included in our analysis.

### Methodological characteristics and quality of studies

All six of the included studies were retrospective in nature [29–34]. Table 1 summarises the methodological characteristics of the included studies. Data quality assessed using the RQS and QUADAS-2 tools was generally satisfactory. All studies were deemed low risk of bias as assessed by the QUADAS-2 tool, whilst 83% (*n* = 5/6) of included studies received an RQS score > 30%. A detailed explanation of the tools and breakdown of the results can be found in supplementary material (S2–S5).

**Table 1:** Study characteristics

Study	Country	Journal	Impact Factor	Primary outcome
Chen [29]	China	European Journal of Radiology	4.531	Progression-free survival
Li [30]	China	American Journal of Roentgenology	6.582	Recurrence-free survival
Li [31]	China	Journal of Oncology	4.501	Disease-free survival
Wang [34]	China	Journal of Ovarian Research	4	Disease-free survival
Wei [32]	China	Frontiers in Oncology	5.738	Recurrence at 18 and 36 months
Wu [22]	China	Abdominal Radiology	2.886	Progression-free survival

### Participant characteristics

The total number of participants from the thirteen included studies was 952. Five studies included both training and validation sets within their studies, whereas the remaining one study included only a training cohort. Overall, 548 patients constituted the training sets and 404 constituted the validation sets across included studies. Of the five studies incorporating validation sets, three were internal [31, 33, 34] and two were external [29, 32]. Basic participant characteristics are outlined in Table 2.

**Table 2:** Participant characteristics

Study	No. Patients		Age (mean ± SD)		Subtype	Treatment received
	T	V	T	V		
Chen [29]	177	77	Recurrence: 50.4 ± 8.1 Non-recurrence: 52.6 ± 8	Recurrence: 50.6 ± 7.6 Non-recurrence: 53 ± 7.8	HGSOC	PDS + AC
Li [30]	117	–	Recurrence: 54.6 ± 8.4 Non-recurrence: 57 ± 7.3		HGSOC	PDS + AC
Li [31]	98	43	56.8 ± 6.2		HGSOC	NAC or PDS + AC
Wang [34]	130	56	47.6 ± 13.4	47.8 ± 12.9	EOC	PDS + AC
Wei [32]	50	Int: 50 Ext: 42	50 ± 3.4	Int: 50 ± 3.2 Ext: 50 ± 3.6	HGSOC	PDS + AC
Wu [22]	74	36	56.7 ± 10.2		HGSOC	PDS + AC

*T* training set, *V* validation set, *HGSOC* high-grade serous ovarian cancer, *EOC* epithelial ovarian cancer, *PDS* primary debulking surgery, *AC* adjuvant chemotherapy, *NAC* neoadjuvant chemotherapy

### Acquisition parameters

Magnetic resonance imaging (MRI) and computed tomography (CT) were the imaging modalities employed by three studies each. Full acquisition parameters are illustrated in Table 3.

**Table 3:** Scanning parameters

Study (CT)	Phase	Model	TV	TC	BP	ST	SI	Matrix	Contrast
Chen [29]	Portal venous	Philips Brilliance 6, NeuViz 128 1.0	120kVp	100–500 mA	0.9, < 1	1–2 mm	0.5–1 mm	512z512	Iohexol 80-100 ml 2.5–3.5 ml/s
Wei [32]	Arterial, venous	Philips Brilliance 6, Philips Brilliance 16, GE Discover CT GE Lightspeed VCT	120kVp	100–500 mA	0.9, < 1	2–5 mm	1.2–1.5 mm	-	Iopamidol 370 80-100 ml, Ultravist 370 60-100 ml
Wu [33]	Arterial, portal venous	Siemens Magnetom Verio	120kVp	350 mA	–	5 mm	5 mm	512 × 512	Contrast medium 80-100 ml 2.5–3.5 ml/s

Study (MRI)	Phase	Model	Field strength	FOV	TE/TR	ST	SI	Matrix	Contrast
Li [30]	Axial FS-T2WI ce-T1W1	Siemens Magnetom Skyra, Magnetom Verio Signa Horizon HDx, Signa Voyager	1.5–3.0 T	300–400 × 390–400	–	3–7 mm	–	160–384 × 320–384	Magnevist 0.1 mmol/kg 2 ml/s
Li [31]	Axial ce-T1W1 Axial FS-T2WI DWI	GE Signa HDXT	3.0 T	240–320 × 240–320	7.5–72.5/500–5000	6 mm	2 mm	96–352 × 130–256	Omniscan 0.1 mmol/kg
Wang [34]	T1WI T2WI ce-T1W1 DWI	Magnetom Avanto	1.5 T	300–380	2.38–83/4.89–8000	3–5 mm	0.9–1.5 mm	–	–

*CT* computed tomography, *TV* tube voltage, *TC* tube current, *BP* beam pitch, *ST* slice thickness, *SI* slice interval, *TE* echo time, *TR* repetition time, *FS* fat suppressed, *CE* contrast enhanced

### Development of signatures

Whilst precise radiomic feature extraction methods varied across included studies, a relatively similar workflow was followed across the board. Experienced radiologists performed manual segmentation of regions of interest (ROI) using ITK-SNAP software in all studies. Features were subsequently extracted from these regions of interest using radiomics software. Specific software utilised in each study is demonstrated in Table 4. Intra- and inter-observer variability were assessed for using the intraclass correlation co-efficient (ICC) in three studies [22, 29, 31]. Acceptable ICC values ranged from 0.75 to 0.85. Four studies reported imaging normalisation methods to account for variation in acquisition parameters [29, 31, 32, 34]. Feature reduction and selection are shown in Table 5.

**Table 4:** Software and performance

Study	Segmentation software	Radiomics software	Performance of signature (Training)			Performance of signature (Validation)		
			AUC	Sens	Spec	AUC	Sens	Spec
Chen [29]	ITK-SNAP v.3.6.1	Intelligence Foundry v3.0.3	0.749 (0.678–0.821)	0.671	0.698	0.769 (0.662–0.877)	0.531	0.911
Li [30]	ITK-SNAP v3.8.0	PyRadiomics v.1.3.1	0.86 (0.78–0.92)	–	–	–	–	–
Li [31]	ITK-SNAP 3.8.0	PyRadiomics	0.83 (0.77–0.90)	0.73	0.81	0.78 (0.65–0.90)	0.80	0.74
Wang [34]	ITK-SNAP	PyRadiomics	0.866 (0.792–0.931)	0.526	0.892	0.818 (0.691–0.932)	0.526	0.892
Wei [32]	ITK-SNAP	MATLAB R2016a	–	–	–	18 m: 0.84 (0.81–0.88) 36 m: 0.89 (0.67–0.96)	–	–
Wu [22]	ITK-SNAP v3.8.0	PyRadiomics	0.899 (0.831–0.966)	0.774	0.884	0.815 (0.668–0.962)	0.929	0.682

18 m: Risk of recurrence at 18 months. 36 m: Risk of recurrence at 36 months

**Table 5:** Feature processing and nomogram construction

Study	Pre-processing	Feature selection process	Selected features	Nomogram
Chen [29]	ZSS IBSI	SCC, SVM-RFE Logistic regression	PLBP_hist_tumor_orient7_0, WL_lbp_hist_cH1_5, PLBP_hist_tu- mor_orient6_2, WL_lbp_hist_cH1_3, original_shape_surfaceArea, origi- nal_glszm_ZoneVariance and original_firstorder_TotalEnergy	Radscore + ICRF (FIGO stage, residual disease)
Li [30]	–	LASSO SMOTE	–	Radscore + ICRF (CA-125 level and residual disease)
Li [31]	B-spline interpolation	Correlation-XX LASSO	wavelet LHL glcm, original shape flatness, wavelet LLH first order kurtosis, original shape flatness, wavelet llh glcm idn, original glcm mcc, original glszm small area low grey-level emphasis	Radscore + ICRF (CA-125, HE4, neutrophil:lymphocyte ratio, FIGO stage, ER/PR positivity, peritoneal mets)
Wang [34]	LoG filters Wavelet filters ZSS	PCC KWT SVM-RFE Logistic regression Random forest	–	Radscore + ICRF (CA-125, Ki67, FIGO)
Wei [32]	Pixel spacing Slice thickness	LASSO Non-zero coefficients COX model	zone-size variance GLSZM, CoifletLLL wavelet transform, FOS feature, small zone low grey-level emphasis GLSZM	Radscore + ICRF (age, FIGO stage, CA-125, residual disease, location, menopause status)
Wu [22]	–	LASSO Ten-fold cross-validation AIC	–	Radscore + ICRF (FIGO, menstruation status, location)

*ZS*: Z-score standardisation, *IBSI* image biomarker standardisation initiative, *LoG* Laplacian of Gaussian, *SCC* spearman correlation co-efficient, *SVM* support vector machine, *RFE* recursive feature elimination, *LASSO* least absolute shrinkage and selection operator, *SMOTE* synthetic minority oversampling technique, *PCC* Pearson correlation co-efficient, *KWT* Kruskal–Wallis test, *AIC* Akaike information criteria, *ICRF* independent clinical risk factors, *FIGO* international federation of gynaecology and obstetrics

### Performance of signatures

Performance of models estimated using the receiver operating characteristic (ROC) curve and summarised as the area under curve (AUC) ranged between studies. Table 4 illustrates the performance of each model in predicting primary outcome. All included studies had at least satisfactory performance in predicting post-operative recurrence. Three studies reported a comparison of model performance between the radiomics/combined model and clinical model alone. All three studies reported a substantial improvement in performance on incorporation of radiomics features into the developed nomogram. [29–31]

## Discussion

Our review demonstrates the development and performance of radiomic-based nomograms to predict post-operative recurrence in patients with ovarian cancer from raw radiological imaging. All nomograms developed within included studies predicted their primary outcome with reasonably modest accuracy (AUC range 0.77–0.89 in validation sets). When assessing the quality of the included studies using the QUADAS-2 and RQS tools, we found that overall, the methodological quality and risk of bias were satisfactory. The studies were generally deemed low risk of bias according to the QUADAS-2 tool and a majority of studies received a high RQS score, indicating good reporting and methodological quality.

Although these findings show promise, heterogeneity amongst studies poses challenges for replication of results. To enable controlled external validation across different institutions, standardisation is necessary, including the use of open-source scans, segmentations, and code [26]. This standardisation will facilitate the eventual application of radiomics within the clinical setting [27, 35]. Our findings also suggest that the quality of the included studies in our review compares favourably to other similar radiomics research, providing confidence in the reliability of the reported results. [36–38]

In the broader context of radiomics research, there are several limitations that need to be acknowledged. One major limitation is the reliability and reproducibility of radiomic features, as they can be influenced by various factors, such as image acquisition protocols and segmentation variability [35]. Standardisation of imaging protocols and rigorous quality control measures are crucial to mitigate these limitations and ensure the reliability of radiomic features. Another challenge is the heterogeneity of radiomics workflows and feature extraction methods across different studies, making direct comparisons and meta-analyses challenging [16]. Efforts towards standardisation and the development of guidelines for radiomics research are essential to address these issues.

Regarding our own study, we acknowledge some minor limitations. Firstly, the limited number of included studies and their retrospective nature might introduce selection bias and impact the generalisability of the findings. Additionally, the variations in study designs, imaging modalities, and analysis techniques amongst the included studies may have influenced the overall results and their interpretation. The authors also understand the concern about potential selection bias due to the inclusion of studies only from one country. Upon analysis of the data, it is evident that all included studies in our systematic review originate from China. Whilst this may raise concerns about generalisability, it is essential to consider the available evidence within the context of the current literature landscape. Despite the geographical concentration of studies, the consistent performance of radiomics-based signatures across different cohorts within these studies suggests promising predictive ability. However, caution should be exercised in extrapolating these findings to diverse patient populations until further validation studies from other regions are conducted. Similarly, negative results may be difficult to publish, further contributing the potential publication bias. Future research efforts should aim to explore the applicability and generalisability of radiomics in predicting disease recurrence in ovarian cancer across various geographical settings.

The potential implications of a radiomics-based signature in clinical practice for predicting post-operative recurrence in ovarian cancer are significant [39]. Such a signature could provide clinicians with a reliable tool to assess individual patient risk and tailor treatment strategies accordingly [40]. The incorporation of radiomic signatures into a nomogram, combined with traditional clinical parameters, has the potential to enhance the accuracy of risk prediction and facilitate personalised treatment decision-making [41]. However, the implementation of radiomics-based nomograms in routine clinical practice may face challenges, including the need for standardised imaging protocols, robust feature extraction and selection methods, and validation prospectively in large-scale multicentre studies. [42–44]

## Conclusion

Our review provides good evidence supporting the potential of radiomics as a predictor of disease recurrence in ovarian cancer. The included studies consistently demonstrated the ability of predicting post-operative recurrence, indicating the potential value of radiomics-based nomograms in improving risk stratification and guiding personalised treatment decisions. However, further research is warranted to validate its real-world benefit in terms of decision-making and patient selection to improve overall outcomes.

## Supplementary Information

Below is the link to the electronic supplementary material.

Supplementary file1 (DOCX 55 KB)

## References

[1] Momenimovahed Z, Tiznobaik A, Taheri S, Salehiniya H. Ovarian cancer in the world: epidemiology and risk factors. *Int J Womens Health.* 2019;11:287-299. 31118829 10.2147/IJWH.S197604 PMC6500433

[2] Brucks JA. Ovarian cancer. The most lethal gynecologic malignancy. *Nurs Clin North Am.* 1992;27(4):835-845. 1448359 10.1016/S0029-6465(22)02813-4

[3] Garzon S, Lagana AS, Casarin J, et al. Secondary and tertiary ovarian cancer recurrence: what is the best management? *Gland Surg.* 2020;9(4):1118-1129. 32953627 10.21037/gs-20-325 PMC7475365

[4] Chang LC, Huang CF, Lai MS, Shen LJ, Wu FL, Cheng WF. Prognostic factors in epithelial ovarian cancer: A population-based study. *PLoS One.* 2018;13(3):e0194993. 29579127 10.1371/journal.pone.0194993 PMC5868839

[5] Dinca AL, Birla RD, Dinca VG, Marica C, Panaitescu E, Constantinoiu S. Prognostic Factors in Advanced Ovarian Cancer - A Clinical Trial. *Chirurgia (Bucur).* 2020;115(1):50-62. 32155399 10.21614/chirurgia.115.1.50

[6] Fernandez-Garza LE, Dominguez-Vigil IG, Garza-Martinez J, Valdez-Aparicio EA, Barrera-Barrera SA, Barrera-Saldana HA. Personalized Medicine in Ovarian Cancer: A Perspective From Mexico. *World J Oncol.* 2021;12(4):85-92. 34349852 10.14740/wjon1383 PMC8297048

[7] Rizzo S, Manganaro L, Dolciami M, Gasparri ML, Papadia A, Del Grande F. Computed Tomography Based Radiomics as a Predictor of Survival in Ovarian Cancer Patients: A Systematic Review. *Cancers (Basel).* 2021;13(3). 33540655 10.3390/cancers13030573 PMC7867247

[8] Zhou M, Scott J, Chaudhury B, et al. Radiomics in Brain Tumor: Image Assessment, Quantitative Feature Descriptors, and Machine-Learning Approaches. *AJNR Am J Neuroradiol.* 2018;39(2):208-216. 28982791 10.3174/ajnr.A5391 PMC5812810

[9] Tiwari P, Prasanna P, Wolansky L, et al. Computer-Extracted Texture Features to Distinguish Cerebral Radionecrosis from Recurrent Brain Tumors on Multiparametric MRI: A Feasibility Study. *AJNR Am J Neuroradiol.* 2016;37(12):2231-2236. 27633806 10.3174/ajnr.A4931 PMC5161689

[10] Pereira S, Pinto A, Alves V, Silva CA. Brain Tumor Segmentation Using Convolutional Neural Networks in MRI Images. *IEEE Trans Med Imaging.* 2016;35(5):1240-1251. 26960222 10.1109/TMI.2016.2538465

[11] Shui L, Ren H, Yang X, et al. The Era of Radiogenomics in Precision Medicine: An Emerging Approach to Support Diagnosis, Treatment Decisions, and Prognostication in Oncology. *Front Oncol.* 2020;10:570465. 33575207 10.3389/fonc.2020.570465 PMC7870863

[12] Liu Q, Jiang P, Jiang Y, et al. Prediction of Aneurysm Stability Using a Machine Learning Model Based on PyRadiomics-Derived Morphological Features. *Stroke.* 2019;50(9):2314-2321. 31288671 10.1161/STROKEAHA.119.025777

[13] Apte AP, Iyer A, Crispin-Ortuzar M, et al. Technical Note: Extension of CERR for computational radiomics: A comprehensive MATLAB platform for reproducible radiomics research. *Med Phys.* 2018. 29896896 10.1002/mp.13046 PMC6597320

[14] Ger RB, Cardenas CE, Anderson BM, et al. Guidelines and Experience Using Imaging Biomarker Explorer (IBEX) for Radiomics. *J Vis Exp.* 2018(131). 29364284 10.3791/57132-v PMC5908445

[15] Singh G, Manjila S, Sakla N, et al. Radiomics and radiogenomics in gliomas: a contemporary update. *Br J Cancer.* 2021;125(5):641-657. 33958734 10.1038/s41416-021-01387-w PMC8405677

[16] Mayerhoefer ME, Materka A, Langs G, et al. Introduction to Radiomics. *J Nucl Med.* 2020;61(4):488-495. 32060219 10.2967/jnumed.118.222893 PMC9374044

[17] Veeraraghavan H, Vargas HA, Jimenez-Sanchez A, et al. Integrated Multi-Tumor Radio-Genomic Marker of Outcomes in Patients with High Serous Ovarian Carcinoma. *Cancers (Basel).* 2020;12(11). 33212885 10.3390/cancers12113403 PMC7698381

[18] Rundo L, Beer L, Escudero Sanchez L, et al. Clinically Interpretable Radiomics-Based Prediction of Histopathologic Response to Neoadjuvant Chemotherapy in High-Grade Serous Ovarian Carcinoma. *Front Oncol.* 2022;12:868265. 35785153 10.3389/fonc.2022.868265 PMC9243357

[19] Meng Y, Sun J, Qu N, Zhang G, Yu T, Piao H. Application of Radiomics for Personalized Treatment of Cancer Patients. *Cancer Manag Res.* 2019;11:10851-10858. 31920394 10.2147/CMAR.S232473 PMC6941598

[20] Liu S, Sun W, Yang S, et al. Deep learning radiomic nomogram to predict recurrence in soft tissue sarcoma: a multi-institutional study. *Eur Radiol.* 2022;32(2):793-805. 34448928 10.1007/s00330-021-08221-0

[21] Zhu C, Huang H, Liu X, et al. A Clinical-Radiomics Nomogram Based on Computed Tomography for Predicting Risk of Local Recurrence After Radiotherapy in Nasopharyngeal Carcinoma. *Front Oncol.* 2021;11:637687. 33816279 10.3389/fonc.2021.637687 PMC8012724

[22] Wu C, Yu S, Zhang Y, Zhu L, Chen S, Liu Y. CT-Based Radiomics Nomogram Improves Risk Stratification and Prediction of Early Recurrence in Hepatocellular Carcinoma After Partial Hepatectomy. *Front Oncol.* 2022;12:896002. 35875140 10.3389/fonc.2022.896002 PMC9302642

[23] Nougaret S, McCague C, Tibermacine H, Vargas HA, Rizzo S, Sala E. Radiomics and radiogenomics in ovarian cancer: a literature review. *Abdom Radiol (NY).* 2021;46(6):2308-2322. 33174120 10.1007/s00261-020-02820-z

[24] Page MJ, McKenzie JE, Bossuyt PM, et al. The PRISMA 2020 statement: an updated guideline for reporting systematic reviews. *BMJ.* 2021;372:n71. 33782057 10.1136/bmj.n71 PMC8005924

[25] Covidence systematic review software. *Veritas Health Innovation, Melbourne, Australia.*

[26] Whiting PF, Rutjes AW, Westwood ME, et al. QUADAS-2: a revised tool for the quality assessment of diagnostic accuracy studies. *Ann Intern Med.* 2011;155(8):529-536. 22007046 10.7326/0003-4819-155-8-201110180-00009

[27] Lambin P, Leijenaar RTH, Deist TM, et al. Radiomics: the bridge between medical imaging and personalized medicine. *Nat Rev Clin Oncol.* 2017;14(12):749-762. 28975929 10.1038/nrclinonc.2017.141

[28] Schiavo JH. PROSPERO: An International Register of Systematic Review Protocols. *Med Ref Serv Q.* 2019;38(2):171-180. 31173570 10.1080/02763869.2019.1588072

[29] Chen HZ, Wang XR, Zhao FM, et al. A CT-based radiomics nomogram for predicting early recurrence in patients with high-grade serous ovarian cancer. *Eur J Radiol.* 2021;145:110018. 34773830 10.1016/j.ejrad.2021.110018

[30] Li HM, Gong J, Li RM, et al. Development of MRI-Based Radiomics Model to Predict the Risk of Recurrence in Patients With Advanced High-Grade Serous Ovarian Carcinoma. *AJR Am J Roentgenol.* 2021;217(3):664-675. 34259544 10.2214/AJR.20.23195

[31] Li C, Wang H, Chen Y, et al. A Nomogram Combining MRI Multisequence Radiomics and Clinical Factors for Predicting Recurrence of High-Grade Serous Ovarian Carcinoma. *J Oncol.* 2022;2022:1716268. 35571486 10.1155/2022/1716268 PMC9095390

[32] Wei W, Liu Z, Rong Y, et al. A Computed Tomography-Based Radiomic Prognostic Marker of Advanced High-Grade Serous Ovarian Cancer Recurrence: A Multicenter Study. *Front Oncol.* 2019;9:255. 31024855 10.3389/fonc.2019.00255 PMC6465630

[33] Wu Y, Jiang W, Fu L, Ren M, Ai H, Wang X. Intra- and peritumoral radiomics for predicting early recurrence in patients with high-grade serous ovarian cancer. *Abdom Radiol (NY).* 2023;48(2):733-743. 36445408 10.1007/s00261-022-03717-9

[34] Wang T, Wang H, Wang Y, et al. MR-based radiomics-clinical nomogram in epithelial ovarian tumor prognosis prediction: tumor body texture analysis across various acquisition protocols. *J Ovarian Res.* 2022;15(1):6. 35022079 10.1186/s13048-021-00941-7 PMC8753904

[35] Xue C, Yuan J, Lo GG, et al. Radiomics feature reliability assessed by intraclass correlation coefficient: a systematic review. *Quant Imaging Med Surg.* 2021;11(10):4431-4460. 34603997 10.21037/qims-21-86 PMC8408801

[36] Wang Q, Li C, Zhang J, et al. Radiomics Models for Predicting Microvascular Invasion in Hepatocellular Carcinoma: A Systematic Review and Radiomics Quality Score Assessment. *Cancers (Basel).* 2021;13(22). 34831018 10.3390/cancers13225864 PMC8616379

[37] Li Y, Liu Y, Liang Y, et al. Radiomics can differentiate high-grade glioma from brain metastasis: a systematic review and meta-analysis. *Eur Radiol.* 2022;32(11):8039-8051. 35587827 10.1007/s00330-022-08828-x

[38] Di Donato V, Kontopantelis E, Cuccu I, et al. Magnetic resonance imaging-radiomics in endometrial cancer: a systematic review and meta-analysis. *Int J Gynecol Cancer.* 2023;33(7):1070-1076. 37094971 10.1136/ijgc-2023-004313

[39] Panico C, Avesani G, Zormpas-Petridis K, Rundo L, Nero C, Sala E. Radiomics and Radiogenomics of Ovarian Cancer: Implications for Treatment Monitoring and Clinical Management. *Radiol Clin North Am.* 2023;61(4):749-760. 37169435 10.1016/j.rcl.2023.02.006

[40] Cui Y, Zhang J, Li Z, et al. A CT-based deep learning radiomics nomogram for predicting the response to neoadjuvant chemotherapy in patients with locally advanced gastric cancer: A multicenter cohort study. *EClinicalMedicine.* 2022;46:101348. 35340629 10.1016/j.eclinm.2022.101348 PMC8943416

[41] Li C, Yin J. Radiomics Nomogram Based on Radiomics Score from Multiregional Diffusion-Weighted MRI and Clinical Factors for Evaluating HER-2 2+ Status of Breast Cancer. *Diagnostics (Basel).* 2021;11(8). 34441425 10.3390/diagnostics11081491 PMC8395031

[42] Fournier L, Costaridou L, Bidaut L, et al. Incorporating radiomics into clinical trials: expert consensus endorsed by the European Society of Radiology on considerations for data-driven compared to biologically driven quantitative biomarkers. *Eur Radiol.* 2021;31(8):6001-6012. 33492473 10.1007/s00330-020-07598-8 PMC8270834

[43] Shi L, Zhang Y, Hu J, et al. Radiomics for the Prediction of Pathological Complete Response to Neoadjuvant Chemoradiation in Locally Advanced Rectal Cancer: A Prospective Observational Trial. *Bioengineering (Basel).* 2023;10(6). 37370565 10.3390/bioengineering10060634 PMC10295574

[44] Keek SA, Wesseling FWR, Woodruff HC, et al. A Prospectively Validated Prognostic Model for Patients with Locally Advanced Squamous Cell Carcinoma of the Head and Neck Based on Radiomics of Computed Tomography Images. *Cancers (Basel).* 2021;13(13). 34210048 10.3390/cancers13133271 PMC8269129

